# Is Deqi an Indicator of Clinical Efficacy of Acupuncture? A Systematic Review

**DOI:** 10.1155/2013/750140

**Published:** 2013-07-31

**Authors:** Shuo Zhang, Wei Mu, Lu Xiao, Wen-Ke Zheng, Chun-Xiang Liu, Li Zhang, Hong-Cai Shang

**Affiliations:** ^1^Center for Evidence-Based Medicine, Tianjin University of Traditional Chinese Medicine, 312 Anshanxi Road, Nankai District, Tianjin 300193, China; ^2^Tianjin Institute for Clinical Evaluation, Tianjin University of Traditional Chinese Medicine, 88 Yuquan Road, Nankai District, Tianjin 300193, China

## Abstract

*Objective*. Despite the systematic literature review of the current evidence, we aim to answer the question “ is Deqi an indicator of clinical effects in acupuncture treatment?” *Methods*. We systematically searched CNKI, VIP, Wanfang Data, PubMed, Embase, and the CENTRAL for three types of study: (1) empirical research probing into the role of Deqi in acupuncture; (2) mechanism studies examining the effect of Deqi on physiological parameters in animal models and human subjects; (3) clinical studies that compared the outcome of acupuncture with Deqi with that of acupuncture without Deqi. Two reviewers independently extracted data, undertook qualitative or quantitative analysis, and summarized findings. *Results*. The ancient Chinese acupuncturists valued the role of Deqi as a diagnostic tool, a prognosis predictor, and a necessary part of the therapeutic procedure. Findings from modern experimental research provided preliminary evidence for the physiological mechanism that produced Deqi. Few clinical studies generated conflicting evidence of the comparative effectiveness of acupuncture with Deqi versus acupuncture without Deqi for a variety of conditions. *Conclusion*. The current evidence base is not solid enough to draw any conclusion regarding the predicative value of natural Deqi for clinical efficacy or the therapeutic value of manipulation-facilitated Deqi.

## 1. Introduction

Deqi (in Chinese pinyin, literally translated as “arrival of qi”) refers to a composite of sensations felt at the needling site after adequate needle insertion with or without proper manipulation. The production of such a special response of the human body is believed to be based on the flow of qi (energy) along channels referred to as meridians in the body. The term is also known as “needling sensation” in more contemporary textbooks and literatures [1]. Typically, the needling sensation is characterized by specific sensory perceptions such as soreness, numbness, distension, and heaviness. However, perceptions of Deqi vary with recipients, manipulation techniques, and the modes of acupuncture stimulation applied. Less frequently, acupuncture recipients may have feelings of coldness, warmth, itching, aching, or twitching, and such a sensation can sometimes be conducted from the needling site towards a more distant area along the meridian. In the meanwhile, the practitioner feels tenseness, drugging, sinking, and vibrations around the needle tail [2].

According to a study [3] that quantitatively defined the uniqueness of the patient's Deqi sensations, aching, soreness, and pressure were found to be most closely related to acupuncture Deqi, as different from tactile stimulation. However, it was also found that the Deqi sensations were mixed with moderate sharp pain feelings in almost one third of all needling procedures, although the less welcomed sharp pain feelings in the form of stabbing, burning or pricking are generally considered to be the result of inadvertent noxious stimulations, rather than that of adequate needling practice. This difference could be evidenced by the variations identified in hemodynamic response between characteristic Deqi sensations and acute pain in fMRI studies described at the last two lines of Table 1.

Moreover, some of the Chinese acupuncture researchers distinguish between two types of the Deqi phenomenon by the perception of the needling sensation by part of either the recipient or the therapist or that by them both. The former is defined as an “implicit” Deqi experience, and the latter is defined as an “explicit” Deqi experience, primarily for convenience of investigation.

While a few acupuncture practitioners and theorists [33–35] declare the value of evocation of qi for diagnostic, therapeutic, and prognostic purposes, as well as in accurately orientating points and providing guidance for manipulation, others [36] argue that the manifestation of the needling sensation is merely a message sent by the human body saying that it has received external stimulations and that implicit Deqi practices can also be effective. Aiming at the current academic controversy surrounding the relationship between Deqi and therapeutic effects of acupuncture, we conducted a systematic review of three types of study centering on the topic. Adopting an evidence-based approach, we aimed to summarize the current evidence profile for the Deqi phenomenon and explore the possibility of converging to a solid conclusion.

## 2. Material and Methods

### 2.1. The Literature Search

We searched three Chinese and three English electronic databases from their respective inception dates to June, 2013, for relevant studies. These included China National Knowledge Infrastructure (CNKI), VIP Data, Wanfang Data, Embase, PubMed, and the Cochrane Central Register of Controlled Trials (CENTRAL). No restriction on the language or the type of publication was made. The Chinese characters used to perform the search included “deqi” (reaching of qi), “qizhi” (arrival of qi), “zhengan” (needling sensation), “zhenci” (acupuncture) and “zhenjiu” (acupuncture and moxibustion), stated here in the Chinese pinyin. English search terms included “deqi”, “de qi”, “acupuncture sensation”, “qi arrival”, “needle sensation”, “needling sensation”, and “needling response”. The references of relevant reviews and the included literatures were checked for possible identification of additional studies.

### 2.2. Study Selection

#### 2.2.1. Inclusion Criteria

In this review, we included the following three types of study:

1. empirical research describing the role of Deqi in acupuncture therapy;
2. research on the physiological mechanisms that produce the Deqi sensation;
3. clinical studies comparing an acupuncture-with-Deqi (hereinafter referred to as AWD) experimental intervention with an acupuncture-without-Deqi (hereinafter referred to as AOD) control.

For the third type of study, we distinguished between two types of the Deqi experience, one being the natural result of needle insertion (defined as natural Deqi) and the other being the product of repeated facilitating manipulations (defined as facilitated Deqi). Based on the result of a pilot search, “natural Deqi” is most frequently viewed by researchers as an exposure in cohort studies, and “facilitated Deqi” is generally considered a part of the interventional procedure in controlled trials. We included both types of the Deqi experience with the aim to have a glimpse of the full picture of the Deqi phenomenon.

For clinical trials, we stipulated that an AWD interventional procedure shall involve intramuscular needle insertion (typically 1-2 cm) followed by manual stimulation until the patient (or the acupuncturist) felt needling sensations and needle retention from 20 to 30 minutes, whereas an AOD control shall be described as either intramuscular or minimal and superficial or subcutaneous needle insertion (typically 0.3 cm) followed by 20 to 30 minutes needle retention without any manipulation. The guidelines for acupoint prescription, treatment session duration, and frequency shall be exactly the same for both groups of intervention in all respects.

#### 2.2.2. Exclusion Criteria

We excluded the studies that focused on acupuncture techniques other than the manual therapy; in view of that, laser or electrical acupuncture may involve quite distinct patterns of stimulation in terms of intensity and frequency. Consequently, the needling sensations elicited and mechanisms of actions could be of sufficient divergence that deserves specialized attention beyond the scope of our study [37, 38]. Clinical studies in which nonpenetrating placebo acupuncture is adopted as the control, such as the Streitberger needle (producing tactile stimuli), or nonacupoints were chosen as the testing sites were excluded for better control of the confounding factors and to minimize the difference between the two manipulation methods in comparison, thus allowing us to concentrate on the effects of Deqi.

Two researchers (S. Zhang and W. Mu) independently managed citations identified from the aforementioned literature search using NoteExpress software (version 2.7, AegeanSoft, Beijing, China) [39]. Firstly, duplications were found and eliminated from the initial combination of search results. Secondly, the apparently irrelevant literatures were excluded after reading the titles and abstracts. Thirdly, the full texts of the potentially relevant studies were read, and ineligible studies were ruled out. Help from a third researcher (H.-C. Shang) was sought whenever there was disagreement.

### 2.3. Data Collection and Quality Assessment Tool

Two reviewers (S. Zhang and W. Mu) designed the data extraction sheet and independently extracted data from original studies. General information on the publication year, the disease type, the treatment course, the outcome measurements, and the methodological characteristics of the included clinical studies is collected and crosschecked. We used the Grade Profiler software version 3.2.2 [40] for evaluation of the quality of included clinical studies following instructions described in the Grade Handbook [41]. The quality of evidence generated from these studies was classified into one of the following four grades.

 
*High Quality*. Further research is very unlikely to change our confidence in the estimate of effect.
 
*Moderate Quality*. Further research is likely to have an important impact on our confidence in the estimate of effect and may change the estimate.
 
*Low Quality*. Further research is very likely to have an important impact on our confidence in the estimate of effect and is likely to change the estimate.
 
*Very Low Quality*. We are very uncertain about the estimate.

Any disagreement was resolved by discussion. Information on the testing sites, the instrument used, and the reported results of mechanism research was also collected and summarized. We did not assess the quality of studies providing empirical insights into the relationship between Deqi and acupuncture effects as well as those exploring the physiological mechanisms underlying acupuncture Deqi.

### 2.4. Data Analysis

Where possible, we used Review Manager version 5.2 [42] provided by the Cochrane Collaborations, for data analysis. Pooled analysis was preferred if sufficient data was provided and homogeneity across studies can be met. When meta-analysis was impossible, comparison between groups was performed for individual studies. If original data was reported, both continuous and dichotomous data were extracted and processed to yield a relative effect. For dichotomous data, a summary risk ratio was presented, and for continuous data a mean difference was calculated, both with 95% confidence intervals. In case of original data being ordinal data, we transformed it into dichotomous data and processed accordingly. The summary statistic was also incorporated into the Grade Profiler and was demonstrated in the summary of the findings tables.

## 3. Results

### 3.1. Results of the Literature Search

A total of 10,492 studies (8,188 from CNKI, 219 from VIP, 1,349 from Wanfang, 209 from PubMed, 465 from Embase, and 62 from CENTRAL) were identified through initial electronic searches; 7,504 studies were left after duplicates were eliminated; and 482 studies were identified after a preliminary screening that ruled out apparently irrelevant studies, comments, or review. A second round of screening excluded ineligible studies after reading the full text. Finally, we included 169 studies in this review: 145 in Chinese and 24 in English.

### 3.2. Discussions on Deqi and Acupuncture Effects in Ancient Medical Books

We identified 120 articles discussing the role of  Deqi in acupuncture therapy from a variety of perspectives. The majority of them cited original texts from ancient Chinese acupuncture classics or textbooks and provided a personal interpretation of the old texts. Some of the studies gave detailed accounts of the practitioner's experiences of eliciting the arrival of qi using different manipulation techniques and offered their insights into the interaction between Deqi and clinical effects. These studies formed the empirical evidence base primarily in favor of the decisive or supporting role of Deqi in relation to acupuncture therapeutic effects. In this section, we cited some of the most exhaustively discussed pieces of quotation from ancient Chinese medical books and gave each of them a plain-English explanation.

In ancient China, the ability to evocate the arrival of qi in the meridian was deemed the criterion for assessing the acupuncturists' level of mastery of professional skills. As was recorded in the Spiritual Pivot (pinyin: Ling Shu), the second text of the Yellow Emperor's Classic of Internal Medicine (Huangdi Neijing) states: “Ordinary acupuncturist treats patients by needling acupoints on the limbs and joints, while an experienced practitioner is able to feel and elicit the arrival of qi in the channel;” see [43].

Further elaboration on the significance of Deqi for an acupuncture treatment was found in a later part of the same treatise, in which the author remarked: “No matter how many times you have manipulated, operate until the needling response arrives. Only when it arrives, will acupuncture be effective. This effect, it is said, is as swift as if the winds blow away the clouds and clear the azure sky. These are the Dao of acupuncture” [43]. A similar statement can be found in the first text of the Yellow Emperor's Classic of Internal Medicine, the Basic Questions (Su Wen), which claimed that “Whether you practice deep or superficial needling in local or distant acupoints, what matters to efficacy is the arrival of qi,” [44] indicating the pivotal role of reaching qi in therapeutic effects regardless of needling depth or selection of points. Furthermore, in the Systematic Classic of Acupuncture and Moxibustion (Zhenjiu Jiayi Jing), the Jin Dynasty scholar Huang Fumi described his manipulation method for eliciting Deqi sensation as “Applying superficial needling and keeping the needle within for minutes to weaken and dispel the evil qi. To navigate the flow of spirit and qi till they accumulate around the needle” [45]. From the previous descriptions, it is not hard to tell that evocation of the arrival of qi was recognized as an indispensable part of the whole treatment procedure.

Also, it was asserted that analysis of the unique features of Deqi sensations in different contexts provided valuable information in relation to the nature of the disease and aided in the differentiation of the TCM syndrome patterns. Citing the famous verse Make It Plain (Biaoyou Fu) written by Dou Hanqing, the Yuan Dynasty philosopher and acupuncturist, he was found saying: “When the needle penetrated the skin, it went further into the heaven level (tian bu), human level (ren bu) and earth level (di bu, these levels refer to the three depths of needle insertion) of the fleshy exterior of human body. At this time, one can determine whether the meridian qi was in deficit or in excess by feeling the power of qi flowing beneath the needle tip. Similarly, one can distinguish between the heat or cold patterns of the zang-fu viscera by sensing the tempo of qi traveling” [46].

Moreover, some of these medical works gave insights into the interaction between the speed of qi arrival and the onset of therapeutic effect, as well as the prognostic values of the Deqi sensations. For instance, Yang Jizhou, the Ming Dynasty acupuncture theorist, remarked in the Compendium of Acupuncture and Moxibustion (Zhenjiu Dacheng) that “Therapeutic effects closely follow the arrival of qi. If the qi comes sooner after the operation, the ailment is easier to cure and the onset of the therapeutic action is rapid. Otherwise, the disease could be hard to cure or even incurable” [46].

A piece of writing collected in the Classic of Difficult Issues (Nan Jing), a medical classic purportedly written by the legendary Chinese physician Bian Que (407–310 B.C.), stated that “If the needle was retained peacefully at the site to await the arrival of qi yet it never came along, it meant the patient has run out of his/her qi in the meridian and faced the imminent danger of death” [47]. Descriptions of the prognostic values of the Deqi sensations perceived by the practitioner were found in a later passage of the Compendium of Acupuncture and Moxibustion, which claimed, “The easier qi arrived, the sooner effects showed off. The needling sensation you perceived can help you predict prognosis. A tightened and dragging needling sensation was indicative of good prognosis, whereas feeling nothing at all indicated poor outcomes” [46].

### 3.3. Studies Exploring the Mechanism Underlying Deqi Sensation

A total of 40 studies were included and reviewed in this section: 20 in Chinese and 20 in English. Generally speaking, we observed a shift of focus on mechanism studies from measuring changes in biochemical parameters at the acupoints before and after the arrival of qi to examining the neural correlates of Deqi sensations using advanced neuroimaging techniques (most frequently fMRI). Two sub-themes were identified. One group of studies tried to explain why the Deqi sensations could be so varied at different points, and the other group examined the Deqi phenomenon at different depths, following varied stimulations, and measured the corresponding sensations produced. All studies were first categorized by themes and then ordered by the experimental model used. The main findings were summarized separately.

Three included studies used animal models to examine the changes in tissue shape or physiological parameters before and after applying AWD. Shi and Zhang [48] found transformation of the subcutaneous connective tissues around the puncturing pore to a whirl as well as dislocated endomysium, vessels, and nerves in the adjacent area, while muscle cells remained intact. Similarly, in an experiment [49] by Langevin and colleagues, significantly thickened layers of subcutaneous connective tissue around the needle and collagen winding along the needle track were found in rats administered AWD, and it was thus hypothesized that mechanical coupling is a mechanism of needle grasp perceived by the acupuncturist. Using self-developed Ca^2+^ selective electrode and push-pull microperfusion technique, Guo et al. [50] compared the impact of AWD on Ca^2+^ distribution at acupoints in a rabbit with that of AWD at adjacent nonacupoints. For the first time, it was found that AWD at an acupoint promoted the redistribution of Ca^2+^ in the body towards aggregation along the same meridian.

Findings of the researches in [4–32, 51–61] exploring the mechanism of acupuncture Deqi sensation in healthy human subjects were summarized in Tables 1, 2, and 3.

### 3.4. Clinical Studies Examining the Correlation between Deqi and Therapeutic Effects

Identified were eight eligible clinical studies evaluating the interaction between Deqi and the therapeutic effects of manual acupuncture for a variety of diseases and conditions. These studies were further divided into two categories: (1) cohort studies observing the predictive value of  Deqi experiences for acupuncture effects and (2) clinical trials testing the comparative effectiveness of AWD versus AODthrough proactively making “qi arrival” happen (by applying manipulations). In two studies [62, 63], medicinal therapy was used in combination with acupuncture, and in one study [64] subcutaneous needle placement was coadministered. Sample sizes ranged from 19 to 338. Details on the general characteristics of the relevant clinical studies were presented in Table 4.

The findings of each clinical study were presented individually as it was impossible to conduct a meaningful meta-analysis with consideration of obvious clinical heterogeneity across the studies. As a result, the credibility of each outcome that involves one single study was assessed using the Grade Profiler. The items “inconsistency” and “publication” were not applicable and thus omitted. The quality of evidence provided by these individual studies was graded from “very low” to “high.” 

#### 3.4.1. Cohort Studies Shed Lights on the Predictive Value of Deqi for Therapeutic Effects

Despite the continuous efforts, we identified only one study [65] in which patients were grouped in terms of whether they naturally experienced Deqi sensations after being acupunctured at Quchi point (LI11). Only one treatment session involving ventricular needle insertion at a depth of 3 cm, remaining of the needle for 30 minutes, and needle removal was administered on 293 patients with primary hypertension. Of the 164 patients having Deqi, 110 were given further stimulations such as needle twirling or rotating in the following 30 minutes, and only 54 were left unintervened until the end of the treatment. Therefore, we gathered the original data of the 129 patients perceiving no natural Deqi and of the 54 patients having Deqi sensations but left unmanipulated to study “whether natural Deqi is predictive of acupuncture efficacy” or “whether natural Deqi is an indicator of better efficacy in comparison with non-Deqi.”

It was found for both groups of patients that blood pressure levels, either systolic pressure (SP) or diastolic pressure (DP), were reduced after treatment (measured before needle insertion and upon needle removal). However, the effect on the AOD group was not clinically significant (mean = −3.232 and SD = 0.963 for SP; mean = −1.132 and SD = 0.747 for DP). The natural AWD group experienced remarkably decreased blood pressure compared with the AOD group (MD = −15.88, 95% CI (−16.34, −15.42) for SP; MD = −6.42, 95% CI (−6.74, −6.10) for DP). In summary, evidence of very low quality showed that, although AOD can change the readings of blood pressure, only Deqi serves to predict clinically relevant effects and is an indicator of greater efficacy (Table 5).

#### 3.4.2. Clinical Trials Intended to Verify Whether AWD Is Superior to AOD in Attaining Efficacy

A total of seven controlled clinical trials were identified. Involving an AWD and an AOD group, they addressed the question “could evocating Deqi sensations facilitated by needling manipulation be a key procedure contributing to the acupuncture effects?” We classified them into two groups according to the type of target disease. Two trials investigated acupuncture for Bell's palsy, and five studies were concerned with various pain conditions. A summary of findings from the two groups of studies was presented separately in Tables 6 and 7.

Mei et al. [62] compared the effects of AWD and AOD, both combined with conventional western medication (prednisone, vitamins B1 and B12, and mecobalamin), on inpatients with Bell's palsy. Outcome measurement was effective rate based on subjective assessment of patient improvement on the House-Brackmann scale (HBS). For ease of comparison, the risk ratio for effective rate was calculated, and no statistical difference was observed between the two groups (RR = 1.40, 95% CI (1.00, 1.97)). This showed that, with western drug being the basic therapy, AWD had no better effects than AOD in terms of improving facial nerve function; however, evidence for generating this conclusion was graded very low in quality; hence, the finding is questionable.

Xu et al. [63] also investigated the comparative effects of AWD versus AOD for Bell's palsy, with prednisone as the basic treatment for both groups. We calculated the risk ratio for complete recovery rate (number of full recoveries/total patient number) using the MH fixed-effect model. Incorporating it (RR = 1.27, 95% CI (1.14, 1.42)) into the Grade system, high-quality evidence showed that AWD helped a moderately greater number of patients make full recovery than AOD. Furthermore, the AWD group attained even greater complete recovery rate (OR = 4.16, 95% CI (2.23, 7.78)), became less facially disabled on facial disability index (FDI) (DLSM (differences of least squares means) = 9.80, 95% CI (6.29, 13.30)), and enjoyed better quality of life measured with WHO Qol-bref (DLSM = 29.86, 95% CI (22.33, 37.38)) at six months following treatment, adjusted for age, sex, treatment center, interval between onset of disease and session commencement, and baseline scores. Logistic regression analysis of a subset of patients (262/338) who completed the Deqi sensation scores (DSS) on a visual analog scale (VAS) showed that higher DSS was slightly predictive of improved facial nerve function (grade-one scores on the HBS) (adjusted OR = 1.07, 95% CI (1.04, 1.09)).

Lund et al. [66] compared the effects of AWD versus AOD on pelvic pain in 70 women in late pregnancy. After 10 treatment sessions, participants in both groups exhibited marked systematic group changes towards lower levels of pain intensity at rest and during routine activities, and in emotional responses and energy losses. However, the same pattern of change in pain intensity and resembling proportions of participants reporting decreased pain were observed, and it was concluded that no difference in effect exists between groups. We performed a secondary data analysis for the primary outcome (pain intensity at rest) and described the intervention effects with the more easily interpretable risk ratios. Originally, pain intensity at rest in the morning and evening was rated on an ordinal scale (VAS), and the change in score was classified into “lower” “unchanged”, and “higher” groups. We defined the “lower” category as the event and calculated the ratio of events for each group (effective rate). Similarly, low-quality evidence indicated that AWD had no better effects than AOD for both outcomes (RR = 0.99, 95% CI (0.69, 1.41) for pain intensity at rest; RR = 1.06, 95% CI (0.73, 1.54) for pain intensity during activities).

Haker and Lundeberg [67] performed a comparative study of AWD and AOD for lateral epicondylalgia. In this study, significant differences were observed between the two techniques immediately following 10 treatment courses in relation to patient-reported recovery (subjective outcome) and pain threshold on gripping and lifting (objective outcome), but such differences disappeared at the 3-month or the 1-year followups. For the subjective outcome, we transformed ordinal data into dichotomous data and calculated the risk ratio using the previously mentioned method. It was found patients receiving AWD reported markedly less elbow pain (RR = 1.35, 95% CI (1.05, 1.73)), but the evidence was low in quality. The AWD group also exhibited enhanced pain-free grip strength and lifting strength in the vigorimeter test and the 3 kg lifting test (*P* < 0.05), respectively. It was then concluded that AWD is superior to AOD in the short-term symptomatic alleviation of elbow pain.

Xiong et al. [68] investigated the effects of acupuncture on primary dysmenorrhea and the correlation of Deqi with such effects. We used the MH fixed-effect model to calculate the risk ratio for effective rate of AWD to AOD in terms of clinical symptom improvement; the criteria for judging “full”, “partial,” “slight,” or “no” recovery were based on the assessment tool described in the Guidance on Practices in Clinical Research of  TCM for Dysmenorrhea [71]. Moderate evidence demonstrated that women in the AWD group experienced significantly greater overall recovery compared with the AOD group (RR = 2.24, 95% CI (1.51, 3.32)). Moreover, they also had an average of 2.78 points greater reduction in pain intensity on the 0–10 VAS (MD = −2.78, 95% CI (−3.61, −1.95)) and had further shortened pain duration (*P* < 0.001). Logistic regression analysis indicated certain correlation between the Deqi sensation scores and the analgesic effects of acupuncture (*R *= 0.654, *P* < 0.001). In a later paper [69] by the same author, data of 30 additional participants was added in the analysis. We chose to report the findings of this study because original data was lacking in the latter paper, but it is worth mentioning that Xiong and colleagues further identified stronger correlation between the acupuncture therapeutic effects and Deqi sensation scores than between efficacy and psychological factors (belief, nervousness, depression, etc.).

Chen [64] reported a trial comparing the analgesia effects of AWD with those of AOD combined with intradermal needle placement for neck pain. The patient-reported Northwick Park questionnaire (NPQ) and the McGill pain questionnaire (MPQ) were collected after the fifth session, upon treatment (ten sessions) completion, at 1- and 3-month followup. For the former outcome, patients in the AWD group experienced greater alleviation of neck pain-associated conditions at treatment conclusion (MD = −17.86, 95% CI (−23.65, −12.07)), and they remained in such a good state after three months (MD = −20.30, 95% CI (−25.32, −15.28)). With regards to the more general feelings of pain, the AWD group perceived less intense pain sensations compared with the AOD group both after ten sessions and at 3 months (MD = −7.80, 95% CI (−10.30, −5.30); MD = −9.06, 95% CI (−11.19, −6.93)). Despite promising results, the strength of this conclusion as an evidence was weakened by imbalanced baseline. It was reported that the AWD group had higher mean NPQ and MPQ scores at baseline, indicating worse pain conditions, and the difference was statistically significant. Thus, it may introduce the thoughts that the AWD group had greater analgesic effects because the more pain-enduring patients were more likely to exhibit improvement.

The last included and most recent study is about AWD versus AOD for migraine by Zheng [70]. A selection of outcomes was discussed here as the trialists used a self-devised migraine assessment tool which lacked validation and calculated response rate in an uncommon way. Patient-important outcomes such as total migraine length and total pain intensity scores (measured with VAS) were measured every four weeks during the eight-week treatment and at 1- and 2-month followups. It was observed that the AOD group generally had a 19.33-hour reduction greater than the AWD group in migraine length per four weeks after treatment (MD = 19.33, 95% CI (9.19, 29.47)); however, no difference between the two groups was detected at 1 and 2 months of followup (MD = 5.41, 95% CI (−4.45, 15.27); MD = 5.90, 95% CI (−4.24, 16.04)). Patients in the AOD group also experienced better pain relief in that total pain score after treatment was 7.01 points lower on average than the AWD group (MD = 7.01, 95% CI (2.80, 11.22)); again such differences disappeared 1 and 2 months later (MD = 0.52, 95% CI (−3.92, 4.96); MD = −5.05, 95% CI (−9.97, −0.13)). In this study, preliminary findings based on data analysis of 19 patients showed that AOD had better short-term analgesic effects than AWD on migraine. However, this evidence was rated very weak in strength as further data analysis including more patient statistics is very likely to change the results.

## 4. Discussions

Acupuncture is an integral component of the traditional Chinese medicine. Since ancient times, the unique phenomenon of Deqi had been observed and widely illustrated in medical books on acupuncture and moxibustion. Recent years have witnessed growing academic interests in the mechanism and utility of Deqi sensations. However, in the past, the exquisite delicacy of Deqi experiences could only be imaged in poetic languages such as in the Make It Plain verse, which stated: “If your feelings are gentle, smooth and slow, the qi has yet to come. When it came, you perceived heaviness, tenseness and unsmoothness underneath the needle. The arrival of qi feels like a fish just swallowed the bait. It sinks and surfaces. When it did not come, you may sense the emptiness as calm and lonely as you were standing in a secluded hall” [46]. Now with the development of acupuncture theories and advanced techniques such as fMRI, a preliminary attempt has been made to reveal the biochemical and physiological basis for the production of the Deqi sensation. Constant efforts have also been made to quantify insertion depths, stimulation intensity, and manipulation procedures, and other factors are believed to have contributed to the effect of Deqi [72]. However, the current evidence profile is insufficient to draw any well-argued conclusion, and a clear mechanism underlying the Deqi sensation remains to be clarified.

In this review, we found that few cohort studies were designed to examine whether Deqi could be a predicator of greater acupuncture efficacy, and evidence generated from controlled clinical trials that can answer the question of “whether manipulation-facilitated AWD is superior to AOD for therapeutic purposes” is also insufficient to come to any solid conclusion. Specifically, one cohort study provided low-quality evidence for the natural emergence of Deqi sensations following needle insertion as an indicator of greater reduction on blood pressure in patients with primary hypertension. Considering AWD versus AOD for Bell's palsy, very low- or high-quality evidence drawn from the two studies came to contradictory findings. And for the analgesic effects of acupuncture, moderate-quality evidence supported the more positive role of AWD for primary dysmenorrhea in terms of enhancing overall recovery and reducing pain. However, very low-to-low quality evidence from the other six studies again provided us with only a complex of contradictions concerning the comparative effects of AWD and AOD. Despite that, the results of the correlation analysis reported in a few studies showed that patients with higher Deqi scores experienced better analgesic effects.

## 5. Conclusion

In summary, ancient Chinese acupuncture theorists and practitioners recognized the dominant role of evocation of the arrival of qi in achieving the best clinical effect. Results of mechanism studies provided preliminary scientific evidence for the production and effects of the Deqi sensation. The current evidence from clinical studies was insufficient to prove the interaction between Deqi and clinical efficacy. Continuing efforts are needed to provide both experimental and clinical evidence for the explanation of such a correlation.

## Figures and Tables

**Table 1:** A summary of studies on Deqi mechanism in acupuncture.

Study ID	Acupoints	Instrument	Results
Lin 1991 [4]	Acupoints on the human thorax	Voll's electroacupuncture devise and electric resistance tester	The electric resistance at acupoints on the human thorax was not correlated with the existence of Deqi sensations at the same point.
Ma 1998 [5]	NA	NA	It is hypothesized that activation of the stretch-activated ion channels is a mediator of the Deqi sensation and the transduction of stimulation signals.
Huang et al. 2012 [6]	LI3, LI4, LI5, LI11	Speckle laser blood flow scanner	AWD at LI11 increased microvascular perfusion at 3 meridian acupoints.
Watanabe et al. 1994 [7]	LI10	DP1100 system	The latency of the event-related potential triggered triggered by AWD was greater than that by electric stimulation. This showed that AWD may influence CNS functions.
Huang 1999 [8]	ST36	EGEG-2DZ	EGG amplitude and the waveform reaction area in two types of Deqi groups differed greatly from those in AOD control.
Sandberg et al. 2003 [9]	ST36	PPG	AWD markedly increased muscle and skin blood flow compared with AOD.
Zhang et al. 2009 [10]	ST36	CDU	AWD greatly changed hemodynamic parameters of the anterior tibial artery.
Yu et al. 2008 [11]	ST36, LI11	CDU	AWD at both points markedly increased the average displacement of the surrounding connective tissues.
Karst et al. 2003 [12]	LI11	Flow cytometry	AWD significantly increased the respiratory burst of neutrophils and slightly dropped beta-endorphin levels.
Streitberger et al. 2008 [13]	LI4	NA	AWD induced more frequent occurrence of vegetative effects and increased occipital EEG power compared with placebo.
Huang et al. 2009 [14]	PC6	PCS	AWD at PC6 markedly increased TCE values measured at a nonacupoint on the meridian.
Huang et al. 2010 [15]	PC6	PCS	AWD at PC6 markedly increased TCE values measured at two nonacupoints on the meridian and at PC3.
Takamoto et al. 2010[16]	#	Functional near-infrared spectroscopy	AWD decreased oxy-Hb concentration in SMA, pre-SMA, and the anterior dorsomedial prefrontal cortex for all stimulated points.
Zhang et al. 2011 [17]	SJ5	PET	AWD activated BA7, -13, -20, -22, -39, -42, and -45.
Lai et al. 2009 [18]	TE5	PET	AWD markedly activated BA13 and 42 and the left cerebellum compared with sham needling.
Chen et al. 2012 [19]	TE5	SPECT	AWD significantly activated BA6, -8, -19, -21, -28, -33, -35, -37, and -47, parahippocampal gyrus, lentiform nucleus, claustrum, and red nucleus, and it deactivated BA9 and -25 compared with sham needling.
Pan et al. 2008 [20]	SP6	fMRI	AWD activated the cortex, the subcortical limbic system, the cingulated gyrus, the lentiform nucleus, the corpus albicans, and the inferior semilunar lobule, and it deactivated the anterior central gyrus and the anterior cingulate.
Zeng 2009 [21]	SJ5	fMRI	AWD markedly activated BA13, -22, -37, -40, -44, -45, and -47, hippocampus, amygdale, and substantia nigra.
Chen et al. 2011 [22]	LI4	fMRI	AWD activated BA4, -6, -9, -13, -17, -18, -19, -21, -22, -23, -29, -30, -35, -36, -37, -39, -40, -41, -42, -43, -44, and -46, and it deactivated medial frontal gyrus, BA24, and the right superior frontal gyrus.
Fang et al. 2012 [23]	LI4	fMRI	AWD deactivated the right amygdale, the cingulated gyrus, the midbrain, the medial frontal gyrus, and the cuneus gyrus.
Fang et al. 2012 [24]	LR3	fMRI	AWD deactivated the limbic-paralimbic-neocortical network and strengthened the connection of these deactivated brain regions.
Tan et al. 2009 [25]	ST36	fMRI	AWD activated functional areas of the cerebral limbic system and dropped serum gastrin levels.
Zhang 2011 [26]	ST36	fMRI	AWD activated cerebral areas SI and SII, the left temporal cortex, the insular cortex, the motor, and supplementary motor cortices, the cingulated gyrus, the hypothalamus, and the amygdaloid body.
Hu et al. 2012 [27]	ST36	fMRI	AWD deactivated the cerebral limbic system and the functional regions associated with language, cognition, and motor control.
Wu et al. 1999 [28]	LI4, ST36	fMRI	AWD at both points activated the hypothalamus and the nucleus accumbens, and it deactivated the rostral part of the anterior cingulate cortex, the amygdala formation, and the hippocampal complex compared with no such effects from AOD.
Gong et al. 2003 [29]	ST36, ST37	fMRI	AWD at both points activated bilateral cingulated gyrus, insular lobe, superior wall of lateral sulcus, and precentral gyrus. AOD at both points activated the left posterior central gyrus. Different cerebral areas were activated during Deqi and non-Deqi at the same point.
Claunch et al. 2012 [30]	LI4, ST36, LR3	fMRI	AWD at all three points deactivated the right subgenual, the right subgenual cingulate, the right isthmus of the cingulum bundle, and the right BA31.
Asghar et al. 2010 [31]	LI4	fMRI	Marked deactivation of the brain area was observed during Deqi in contrast to the occurrence of a mixture of activations and deactivations in the acute pain group.
Hui et al. 2005 [32]	ST36	fMRI	Attenuation of signal intensity in the limbic and paralimbic structures of cortical and subcortical regions in telencephalon, the diencephalon, the brainstem, and the cerebellum was observed during AWD compared with signal increase with the acute pain and the AOD group.

#: acupoints and nonacupoints within the right extensor muscle in the forearm; AOD: acupuncture without Deqi; AWD: acupuncture with Deqi; BA: brodmann area; CDU: color Doppler ultrasound; CNS: central nervous system; EGG: electrogastroenterogram; fMRI: functional magnetic resonance imaging; NA: not available; PCS: percutaneous carbon dioxide sensor; PET: positron emission tomography; PPG: photoplethysmography; SI: secondary somatosensory cortex; SII: primal somatosensory cortex; SMA: supplementary motor area; SPECT: single-photon emission computed tomography; TCE: transcutaneous CO_2_ emission.

**Table 2:** A summary of studies on mechanisms underlying varied Deqi sensations.

Study ID	Acupoints	Instrument	Results
Bossy et al. 1984 [51]	Jing points at the hand	NA	Deqi resulted from correct stimulation of the various structures in relation to an acupoint, such as group II afferent fibers.
Wang et al. 1985 [52]	PC6, LU11	NA	Numbness and soreness were conveyed by Group II and Group IV fibers, and heaviness and distention by Group III fibers.
Wang and Liu 1989 [53]	PC6, PC9, LI1, LU10, LU11	NA	Needling stimulation primarily activated slowly adapting receptors. The type of receptors varied with the location of acupoints.
Kuo et al. 2010 [54]	LU5, LU7	LDF	Strong Deqi sensations, heat and numbness, felt at LU5 were correlated with increased blood flow at LU5.
Kuo et al. 2004 [55]	SI6, SI8	LDF	AWD increased blood flow at acupoints. The speedy flowing of tissue fluid along the body stalk may explain the occurrence of propagated sensation along the meridian.
Kuo et al. 2004 [56]	LI4, LI11	LDF	Deqi sensations such as soreness, numbness, and heat coexisted with increased blood flow at acupoints.
Lee et al. 2010 [57]	SP3, KI2	Ultrasound dopplerography	Deqi-related warm, radiating, and energetic feelings were correlated with decreased blood flow velocity.
Zhang et al. 2011 [58]	SJ5	fMRI	Deqi sensations perceived at SJ5 were mainly soreness, numbness, distending, and heaviness, corresponding to activated left temporal lobe and superior temporal gyrus. By contrast, tingling was felt at a neighboring nonacupoint, and the left limbic lobe and hippocampal gyrus were excited.

Abbreviations: fMRI: functional magnetic resonance imaging; LDF: laser doppler flowmeter; NA: not available.

**Table 3:** A summary of mechanism studies on needling intensity and Deqi.

Study ID	Acupoints	Instrument	Results
Deng and Zhou 2010 [59]	ST36	PowerLab 4/25	A marked difference was observed in muscular contractility at Deqi depth compared with that at two non-Deqi depths.
Choi et al. 2012 [60]	SP6, SP9, ST36, GB39	SASS	Pressure pain threshold and Deqi sensation increased as acupuncture simulation intensified (needling with rotation > deep needling > superficial needling).
Park et al. 2011 [61]	NA	Ultrasound imaging	Pricking and sharp sensations appeared more frequently when shallower tissues were needled, whereas deep, dull, heavy, spreading, and electric feelings predominated in deeper tissue levels. The introduction of needle rotation in addition to oscillation intensified deep, dull, and heavy rather than pricking and sharp sensations.

Abbreviations: NA: not available; SASS: subjective acupuncture sensation scale.

**Table 4:** Characteristics of the included clinical studies.

Study ID	Type of disease	Sample (T/C)	Comparison (exposure)	Treatment course	Outcome measures
Ma 2012 [65]	Primary hypertension	293 totally	Patient-reported natural Deqi after intramuscular needle insertion versus noncharacteristic Deqi sensations	One 30 m session	Blood pressure
Mei et al. 2010 [62]	Bell's palsy	28/22	Intramuscular insertion and manipulation + medication versus nonmanipulation + medication	Five 30 m sessions per week for 4 weeks	Effective rate based on HBS, 16PF, HAMA, and DSS (VAS)
Xu et al. 2013 [63]	Bell's palsy	167/171 159/157	Intramuscular insertion and manipulation until Deqi + medication versus nonmanipulation + medication	Five 30 m sessions per week for 4 weeks	Facial-nerve function (HBS), FDI, WHO HR-QoL, DSS (VAS), and adverse events
Lund et al. 2006 [66]	Pelvic pain in late pregnancy	35/35 22/25	Intramuscular insertion and manipulation until the patient-reported Deqi versus subcutaneous insertion and nonmanipulation	Two 30 m sessions per week for 5 weeks	Pain intensity (VAS) at rest/during daily activities and NHPQ
Haker and Lundeberg 1990 [67]	Epicondylalgia	86 in total 44/38	Intramuscular insertion and manipulation until Deqi versus subcutaneous insertion and nonmanipulation	Ten 20 m sessions (2 or 3 times weekly), and followup after 3 and 12 months	Patient-reported pain improvement, lifting test, and vigorimeter test
Xiong et al. 2011 [68]	Primary dysmenorrhea	45/45 (67/64, 60/60 for Xiong et al. 2012 [69])	Intramuscular insertion (1-2 cm) and manipulation until Deqi versus nonmanipulation	Five consecutive 30 m sessions per menstrual cycle and for 3 courses	Effective rate, pain intensity (VAS), pain duration, and DSS (nervousness using VAS, acupuncture confidence questionnaire, EPQ, and 16PF were added in Xiong et al. 2012 [69])
Chen 2011 [64]	Cervical spondylosis	36/34	Intramuscular insertion and manipulation until Deqi + intradermal needle placement versus subcutaneous insertion and nonmanipulation + intradermal needle placement	Ten 20 m sessions, and followup at 1 and 3 months	NPQ, MPQ, and SF-36
Zheng 2012 [70]	Migraine	9/10 (completed) Ongoing study	Intramuscular insertion and manipulation until Deqi versus subcutaneous insertion and nonmanipulation	Twelve 30 m sessions, lasting for 8 weeks. Followup at 1 and 2 months	Migraine assessment tool (self-devised), pain intensity (VAS), pain duration, response rate, and safety

The latter set of numbers in the “Sample” column refers to the number of participants included in data analysis. Abbreviations. DSS: Deqi sensation scale. It is a tool providing typical descriptors of the needling sensations for patients to choose from those the best that represent their experience. Combining with VAS, it allows rating of the intensity of response to each sensation ranging from “none” to “unbearable pain,” or on a numeric scale. EPQ: eysenck personality questionnaire; FDI: facial disability index; FDIP: FDI physical function scores; HAMA: the Hamilton anxiety scale; HBS: House-Brackmann scale; m: minute; MPQ: the McGill pain questionnaire; NHPQ: the Nottingham health profile questionnaire; NPQ: the Northwick Park questionnaire; 16PF: 16 personality factor questionnaire; VAS: visual analog scale.

**Table 5:** Summary of the findings table for the evidence of the predicative value of natural Deqi for clinical efficacy.

Natural AWD compared with natural AOD for primary hypertension				
Outcomes	Illustrative comparative risks (95% CI)		No. of participants (studies)	Quality of the evidence (grade)
	Natural AOD	Natural AWD		
Blood pressure (SP) Scale from 0 to 200	The mean systolic blood pressure in the control groups was 152.225 mmHg	The mean systolic blood pressure in the intervention group was 15.88 mmHg lower (16.34 to 15.42 mmHg lower)	183 (1 study)	⊕⊝⊝ ⊝ very low^1,2,3,4^

Blood pressure (DP) Scale from 0 to 200	The mean diastolic blood pressure in the control groups was 93.093 mmHg	The mean diastolic blood pressure in the intervention group was 6.42 mmHg lower (6.74 to 6.10 mmHg lower)	183 (1 study)	⊕⊝⊝ ⊝ very low^1,2,3^

^1^This single cohort study has appropriate eligibility criteria, but it suffers from subjective measurement of exposure (patient-reported Deqi sensation) and very short treatment course (one session and no followup).

^2^Very narrow CI. Confidence interval < 1/10 effect size.

^3^A single study is very likely to be biased.

^4^It was observed that the mean difference of blood pressure was 15.88 lower in AWD group compared with AOD group. The effect size is large.

**Table 6:** Summary of the findings table for the evidence of comparative effects of AWD versus AOD for Bell Palsy.

AWD compared with AOD for Bell Palsy						
Study ID	Outcomes	Illustrative comparative risks (95% CI)		Relative effect (95% CI)	No. of participants (studies)	Quality of the evidence (grade)
		AOD (assumed risk)	AWD (corresponding risk)			
Mei et al. 2010 [62]	Effective rate (followup: 3 months) Assessment of changes in facial nerve functions based on House-Brackmann scale^1^	Study population		RR 1.40 (1.00 to 1.97)	50 (1 study)	⊕⊝⊝⊝ very low^2,3,4,5^
		636 per 1000	890 per 1000 (636 to 1000)			

Xu et al 2013 [63]	Complete recovery rate (followup: 6 months) House-Brackmann score graded by 3 assessors	Study population		RR 1.27 (1.14 to 1.42)	338 (1 study)	⊕⊕⊕ ⊕ high^3,5,6,7^
		708 per 1000	899 per 1000 (807 to 1000)			

^1^Patient-important outcome.

^2^Randomization methods and allocation concealment not mentioned. Stratified and randomized assignment and binding of the patient were mentioned. For acupuncture trials, blinding of the practitioner is impossible. None lost to followup. No selective outcome reporting.

^3^This item was omitted here because we assessed one single study.

^4^Subjective assessment based on any observed improvement on HB scale for facial nerve function. RR has a wide CI; it almost equals effect size and covers 1.0.

^5^A single study is very likely to be biased. However, it was omitted here to avoid all evidence being “very low” in quality and therefore indistinguishable.

^6^Computer-generated random number sequence, randomized assignment, allocation concealment (sealed opaque envelope, and a designated personnel kept it) and blinding of the patient, recruiter, and assessor were described. For acupuncture trials, blinding of the practitioner is impossible; 22/338 dropouts, ITT analysis done. No selective outcome reporting.

^7^Subjective outcome, but rigorously controlled. Specifically, three skilled experts rated scores according to the House-Brackmann scale. For RR, narrow CI equals 1/10 effect size.

**Table 7:** Summary of the findings table for the evidence of comparative effects of AWD versus AOD for pain.

Acupuncture with Deqi versus acupuncture without Deqi for pain						
Study ID and descriptions	Outcomes	Illustrative comparative risks (95% CI)		Relative effect (95% CI)	No. of participants (studies)	Quality of the evidence (grade)
		AOD (assumed risk)	AWD (corresponding risk)			
Lund et al. 2006 [66] Subject: acupuncture for pelvic pain in late pregnancy Setting: two maternity healthcare departments in Sweden	Effective rate for change in morning pain intensity after treatment assessed with change in VAS scores, grouped into “lower,” “unchanged,” and “higher”	Study population		RR 0.99 (0.69 to 1.41)	47 (1 study)	⊕⊕⊝⊝ low^1,2,3,4,5^
		727 per 1000	720 per 1000 (502 to 1000)			
	Effective rate for change in evening pain intensity after treatment assessed with change in VAS scores, grouped into “lower,” “unchanged,” and “higher”	Study population		RR 1.06 (0.73 to 1.54)	47 (1 study)	⊕⊕⊝⊝ low^1,2,3,4^
		682 per 1000	723 per 1000 (498 to 1000)			

Haker and Lundeberg 1990 [67] Subject: acupuncture for epicondylalgia Setting: outpatients in Sweden	Effective rate for patient reported recovery after treatment assessed with a scale from “unchanged”/“worse” to “excellent” recovery	Study population		RR 1.35 (1.05 to 1.73)	82 (1 study)	⊕⊕⊝⊝ low^2,4,6,7^
		658 per 1000	888 per 1000 (691 to 1000)			

Xiong et al. 2011 [68] Subject: acupuncture for primary dysmenorrhea Setting: outpatients from Tongji Hospital, Wuhan, China	Effective rate for pain relief after treatment assessed with the efficacy assessment guideline for TCM for primary dysmenorrhea	Study population		RR 2.24 (1.51 to 3.32)	90 (1 study)	⊕⊕⊕⊝ moderate^2,4,7,8^
		378 per 1000	847 per 1000 (571 to 1000)			
	Pain intensity after treatment assessed with VAS, scale from 0 to 10	The mean pain intensity score in the control group was 4.48	The mean pain intensity score in the intervention group was 2.78 lower (3.61 to 1.95 lower)		90 (1 study)	⊕⊕⊕⊝ moderate^2,4,7,8^

Chen et al. 2011 [22] Subject: acupuncture plus intradermal needle placement for cervical spondylosis Setting: volunteers recruited in two Guangdong hospitals in China	Neck pain after treatment assessed with the Northwick Park questionnaire, scale from 0 to 100	The mean neck pain score in the control group was 23.56	The mean neck pain score in the intervention group was 17.86 lower (23.65 to 12.07 lower)		70 (1 study)	⊕⊕⊝⊝ low^2,4,7,9^
	Pain after treatment assessed with the McGill pain questionnaire, scale from 0 to 60	The mean pain score in the control group was 20.35	The mean pain score in the intervention group was 7.80 lower (10.3 to 5.3 lower)		70 (1 study)	⊕⊕⊝⊝ low^2,4,7,9^

Zheng 2012 [70] Subject: acupuncture for migraine Setting: outpatients from two acupuncture clinics in Beijing, China Note: this study is ongoing by the time of publication, so incomplete data was reported.	Total migraine hours per 4 weeks after treatment	The mean total migraine hours per 4 weeks in the control group were 21.95 hours	The mean total migraine hours per 4 weeks in the intervention group were 19.33 hours longer (9.19 to 29.47 hours longer)		19 (1 study)	⊕⊝⊝⊝ very low^2,7,10,11^
	Migraine pain intensity (total VAS score per 4 weeks) after treatment assessed with VAS, scale from 0 to 10	The mean migraine pain intensity score in the control group was 11.70	The mean migraine pain intensity score in the intervention group was 7.01 higher (2.8 to 11.22 higher)		19 (1 study)	⊕⊝⊝⊝ very low^2,7,10,11^

^1^Randomization method and blinding of the patient not mentioned. Randomized assignment, binding of outcome assessor, and allocation concealment mentioned. For acupuncture trials, blinding of the practitioner is impossible; 3/70 patients were lost to followup; reasons explained; no ITT analysis. No selective outcome reporting.

^2^This item was omitted here because we assessed one single study.

^3^For this single study, findings presented evident individual variations in both groups. The calculated CI equals 1/3–1/2 effect size.

^4^A single study is very likely to be biased. However, it was omitted here to avoid all evidence being “very low” in quality and therefore indistinguishable.

^5^Patient-important outcome.

^6^Randomization methods, allocation concealment, and blinding of the patient not mentioned. Randomized assignment and binding of outcome assessor mentioned. For acupuncture trials, blinding of the practitioner is impossible; 4/86 patients were lost to followup; reasons explained; no ITT analysis. No selective outcome reporting.

^7^Subjective assessments. The calculated CI equals 1/9–2/3 effect size.

^8^Random number table, randomized assignment, allocation concealment, and blinding of the patient and assessor described. For acupuncture trials, blinding of the practitioner is impossible. No dropouts. No selective outcome reporting.

^9^Central randomization, randomized assignment, and the use of sealed envelope described. Placebo acupuncture was used, and the patient was blinded. However, blinding of the outcome assessor was not mentioned. For acupuncture trials, blinding of the practitioner is impossible. It is highly suspected that the physicians act as assessors; hence, the risk for measurement bias is high. No dropouts. No selective outcome reporting. Imbalanced baseline was reported.

^10^Central and block randomization and allocation concealment described. The outcome assessor was blinded, but both the patient and the acupuncturist were aware of the allocation. For acupuncture trials, blinding of the practitioner is impossible. High dropout rate (22/59); reasons explained. No selective outcome reporting.

^11^The trial is ongoing by the time of publication. Preliminary results were published (19/48 cases planned), with high risk of biases.
